# Equity in diagnosis through adequate clinical trial design in diagnostic performance studies

**DOI:** 10.1002/alz70856_102804

**Published:** 2025-12-24

**Authors:** Imke Kirste, David Caley, Clara Quijano Rubio, Margherita Carboni

**Affiliations:** ^1^ Roche Diagnostics, Durham, NC, USA; ^2^ Roche Diagnostics Ltd, Burgess Hill, West Sussex, United Kingdom; ^3^ Roche Diagnostics International Ltd, Rotkreuz, Zug, Switzerland

## Abstract

**Background:**

Access to healthcare is unbalanced across different populations with disparities in the availability of diagnostic tests. Additionally, test development currently relies on studies with limited representation of minorities and comorbidities present in the real clinical population. In order to develop diagnostic tests that can bring medical value to a broad population, clinical trial design needs to take these aspects into consideration.

**Method:**

As part of the development of a novel blood‐based biomarker for patients with suspected early progression towards Alzheimer's disease Roche is currently running a clinical study aiming to include racial and ethnic minorities as well as subjects with comorbidities. Our approach includes the use of mobile units for patient recruitment, multilingual study materials, outreach events, study site selection based on geographic location and proven capability of diverse enrollment. The current clinical trial has a goal of enrollment of 1500 patients into two arms: cut‐off determination and validation.

**Result:**

This approach led to a significant representation of racial and ethnic minorities as well as inclusion of patients with multiple comorbidities, potentially better reflecting the true patient population. Out of 600 enrolled patients in the cut‐off determination arm, 387 (64.5%) are non‐hispanic caucasian. African Americans reflect 12.5% (*n* = 75) of the study population with the remaining minorities including Multiracial and Asian‐Americans. Nearly all of our patients have more than one comorbidity reflective of the target clinical population including cardiovascular, metabolic, neurological, renal, oncological, and immunological conditions as well as other age and non‐age related diseases. Recruitment into the pivotal trial arm for cut‐off validation is still ongoing but shows an even slightly better diversity profile.

**Conclusion:**

A carefully designed clinical trial can achieve valid reflection of real‐world disease population leading to more accurate results and better reflection of post‐launch usage.

